# Biosafety assessment of *Acinetobacter* strains isolated from the Three Gorges Reservoir region in nematode *Caenorhabditis elegans*

**DOI:** 10.1038/s41598-021-99274-0

**Published:** 2021-10-05

**Authors:** Yunjia Deng, Huihui Du, Mingfeng Tang, Qilong Wang, Qian Huang, Ying He, Fei Cheng, Feng Zhao, Dayong Wang, Guosheng Xiao

**Affiliations:** 1 College of Biology and Food Engineering, Chongqing Three Gorges University, Wanzhou, Chongqing, China; 2 Key Laboratory of Water Environment Evolution and Pollution Control in Three Gorges Reservoir, Chongqing Three Gorges University, Wanzhou, Chongqing, China; 3 Engineering Technology Research Center of Characteristic Biological Resources in Northeast Chongqing, Chongqing Three Gorges University, Wanzhou, Chongqing, China

**Keywords:** Bacteria, Bacterial host response, Bacterial immune evasion, Bacterial pathogenesis, Microbiology, Pathogenesis, Microbial ecology

## Abstract

*Acinetobacter* has been frequently detected in backwater areas of the Three Gorges Reservoir (TGR) region. We here employed *Caenorhabditis elegans* to perform biosafety assessment of *Acinetobacter* strains isolated from backwater area in the TGR region. Among 21 isolates and 5 reference strains of *Acinetobacter*, exposure to *Acinetobacter* strains of AC1, AC15, AC18, AC21, *A. baumannii* ATCC 19606^T^, *A. junii* NH88-14, and *A. lwoffii* DSM 2403^T^ resulted in significant decrease in locomotion behavior and reduction in lifespan of *Caenorhabditis elegans*. In nematodes, exposure to *Acinetobacter* strains of AC1, AC15, AC18, AC21, *A. baumannii*, *A. junii* and *A. lwoffii* also resulted in significant reactive oxygen species (ROS) production. Moreover, exposure to *Acinetobacter* isolates of AC1, AC15, AC18, and AC21 led to significant increase in expressions of both SOD-3::GFP and some antimicrobial genes (*lys-1*, *spp-12*, *lys-7*, *dod-6*, *spp-1*, *dod-22*, *lys-8*, and/or *F55G11.4*) in nematodes. The *Acinetobacter* isolates of AC1, AC15, AC18, and AC21 had different morphological, biochemical, phylogenetical, and virulence gene properties. Our results suggested that exposure risk of some *Acinetobacter* strains isolated from the TGR region exists for environmental organisms and human health. In addition, *C. elegans* is useful to assess biosafety of *Acinetobacter* isolates from the environment.

The Three Gorges Reservoir (TGR), whose distance is approximately 662.9 km, is a major water source in China. Water fluctuation in the TGR region produces a water-level fluctuating zone (WLFZ) every year^1^. That is, a novel ecosystem is created by construction of the TGR Dam. Meanwhile, due to rapid development in industrialization and urbanization in the recent years, various organic and inorganic pollutants are potentially released into the environment in the TGR region through industrial or residential wastewater^2–5^. Mover, a large amount of bacterioplankton community, including waterborne pathogens, has been detected in the TGR region^6–8^.

*Caenorhabditis elegans* can be used to perform the toxicological study at the whole animal level^9–12^. *C. elegans* has become an ideal surrogate model to determine both pathogenesis and conserved mechanisms in host-microbe interactions of human pathogens^13,14^. More importantly, *C. elegans* is highly sensitive to various environmental exposures^15–18^. Considering the sensitivity to environmental exposure, it has been employed to perform biosafety evaluation of water samples in TGR region in both flood season and quiet season^19,20^. Based on our previous toxicity evaluation, only acute exposure to water sample in backwater area resulted in toxic effects on nematodes, such as decrease in locomotion behavior and activation of oxidative stress^20^. Moreover, both liquid phase and solid phase contributed to toxicity induction of water sample in backwater area^20^. In the liquid phase, the potential toxicants were suggested to be the organic pollutants^20^. Nevertheless, the toxicity contributors in the solid phase of water sample in backwater area in the TGR region are still largely unclear.

Environmental pathogens are an important component in the solid phase of surface water samples. Existence of high prevalence pathogens has been found in backwater areas of the TGR region^7,8^. Environmental *Acinetobacter* was realized as an important nosocomial pathogen during the late 1970s, and has received an increasing attention because of its potential to cause severe nosocomial infections and formation of multiple-drug and pan-drug resistance^21,22^. *Acinetobacter* occupies a considerable position in nature because it prevails in natural environments, such as soil, oceans, fresh water, and sediments^23,24^. Nevertheless, the biosafety properties of *Acinetobacter* in the TGR region remain largely unclear. We here aimed at performing biosafety assessment of *Acinetobacter* strains isolated from backwater areas in the TGR region in nematodes. Among the isolated 21 *Acinetobacter* strains, exposure to four isolates (AC1, AC15, AC18, and AC21) resulted in toxic effects in nematodes. Our data implied the possible exposure risk of some *Acinetobacter* strains in the TGR region for environmental organisms and human health.

## Results

### *Acinetobacter* isolates from the TGR region

**Figure 1:**
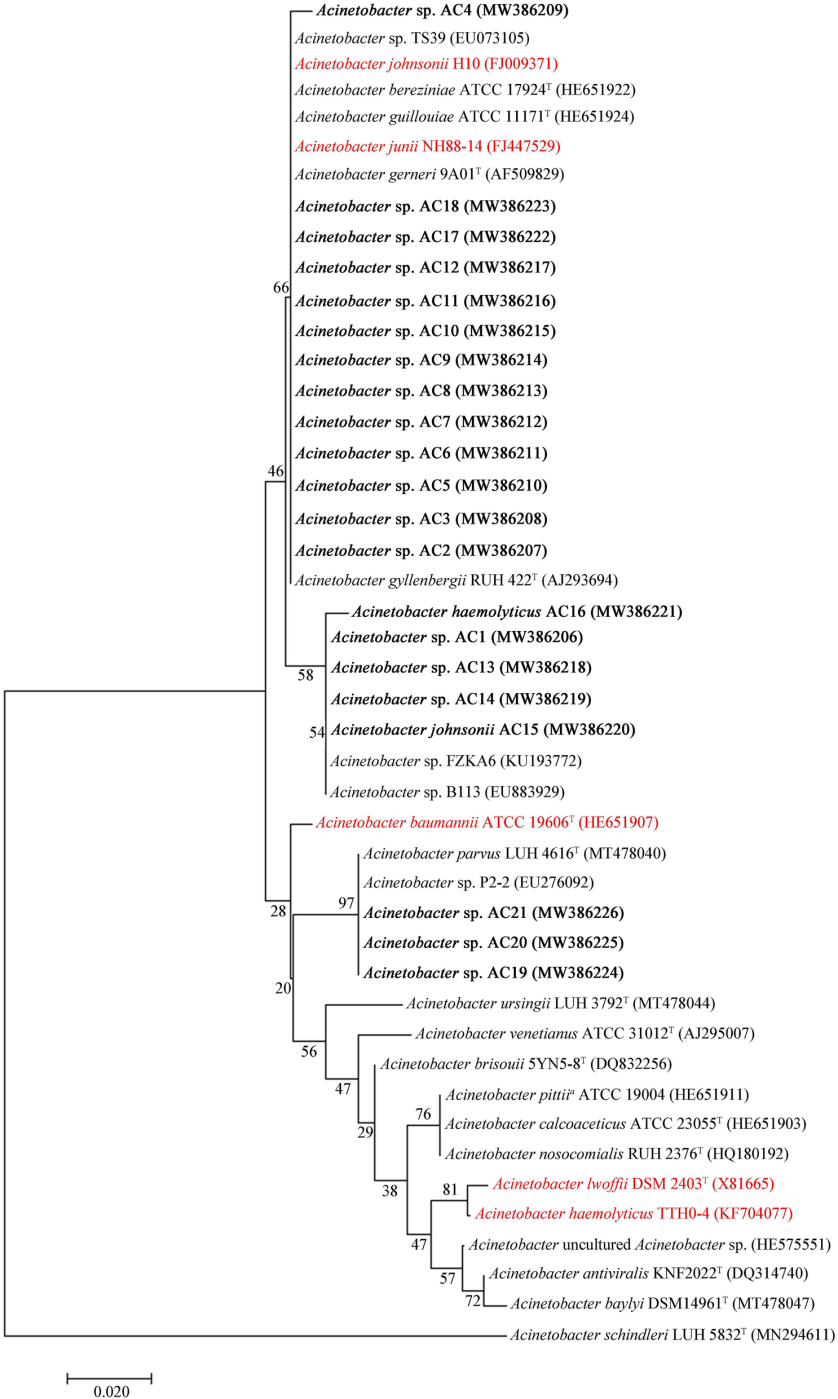
A phylogenetic tree of 16S rRNA gene sequences showing position of isolates among species of genus *Acinetobacter.* Both isolates from the TGR region (the bold fonts) and reference strains used to infect *Caenorhabditis elegans* (the red fonts) are shown.

In the TGR region, we isolated 21 *Acinetobacter* strains (one *A. johnsonii*, one *A. haemolyticus* and 19 *Acinetobacter* sp. strains) (Table S1). Based on phylogenetic analysis after 16S rRNA gene sequencing, these 21 isolates belong to the genus *A*cinetobacter, exhibiting a similarity of 95.38–99.93% with known *Acinetobacter* strains in GenBank (Table S1). In phylogenetic tree (N-J) constructed with both isolated and known *Acinetobacter* strains, these 21 isolates branched deeply with three *Acinetobacter* clusters consisting of important clinical *A*cinetobacter species, such as *A. johnsonii* H10 (FJ009371), *A. junii* NH88-14 (FJ447529), *A. baumannii* ATCC19606^T^ (HE651907), *A. lwoffii* DSM2403^T^ (X81665) and *A. haemolyticus* TTH04-1 (KF704077) (Fig. 1). Five reference *Acinetobacter* strains were selected and used^21^. Currently, the genus *Acinetobacter* comprises 68 species with validly-published names (https://apps.szu.cz/anemec/Classification.pdf, May 25, 2021). Among the named species, *A. baumannii* is the most studied species associated with clinical infections followed by the non-*A. baumannii* species *A. haemolyticus*, *A. junii*, *A. johnsonii*, and *A. lwofii*^21^.

### Effect of different *Acinetobacter* strains isolated from the TGR region and reference strains on lifespan of nematodes

**Figure 2:**
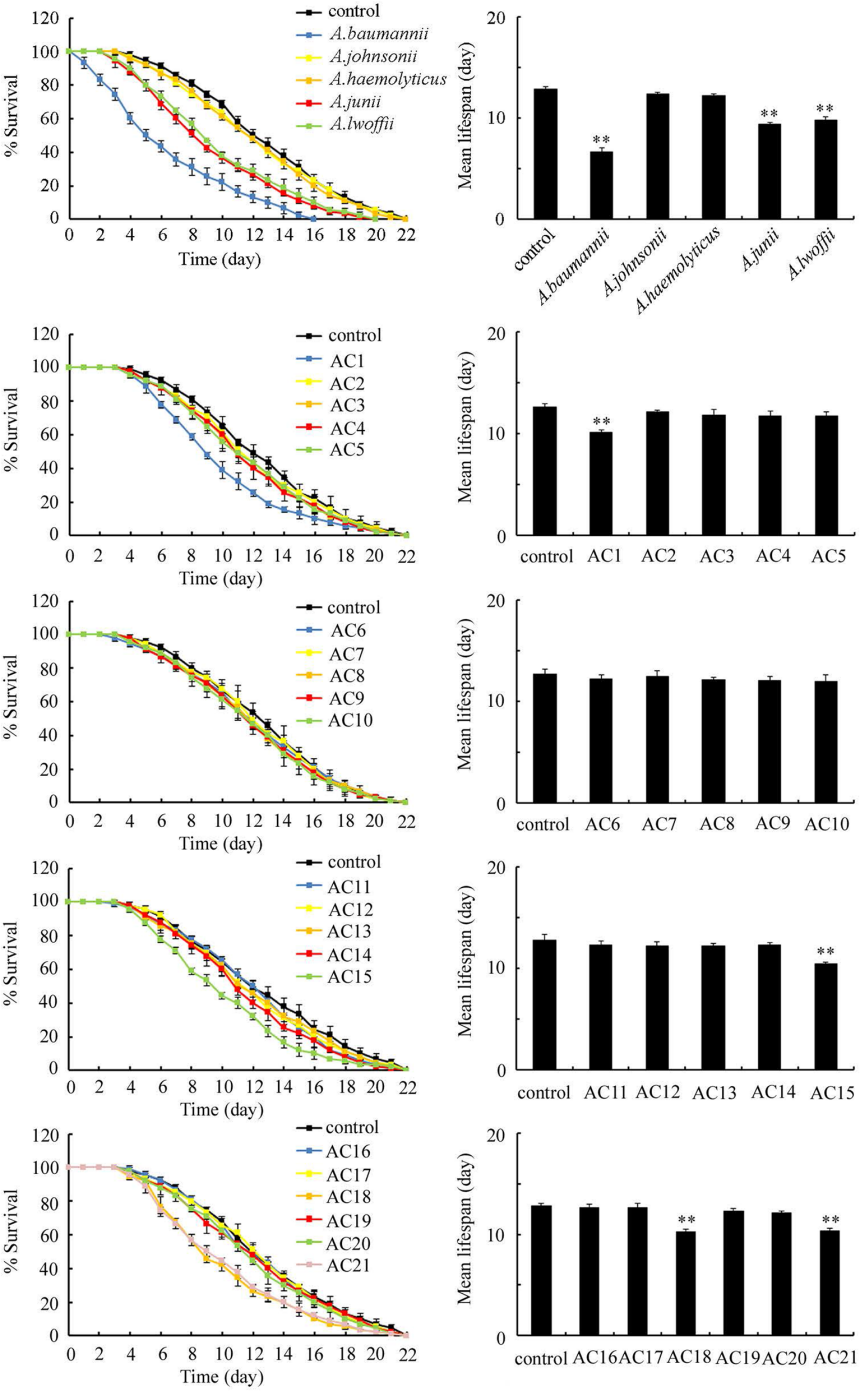
Effect of exposure to different *Acinetobacter* strains isolated from the TGR region and reference strains on lifespan in wild-type nematodes. The L4-larvae nematodes were exposed to *Acinetobacter* for 24-h. Control, unexposed nematodes. Bars represent means ± SD. **P < 0.01 versus control.

L4-larvae were exposed to different *Acinetobacter* strains for 24-h. Totally 21 *Acinetobacter* strains isolated from the TGR region and 5 reference strains of *Acinetobacter* species were used for the lifespan analysis. Based on the comparison of lifespan curves, exposure to *Acinetobacter* strains of AC2, AC3, AC4, AC5, AC6, AC7, AC8, AC9, AC10, AC11, AC12, AC13, AC14, AC16, AC17, AC19, AC20, *A. johnsonii* H10, and *A. haemolyticus* TTH0-4 could not alter lifespan curve (Fig. 2). Similarly, *Acinetobacter* strains of AC2, AC3, AC4, AC5, AC6, AC7, AC8, AC9, AC10, AC11, AC12, AC13, AC14, AC16, AC17, AC19, AC20, *A. johnsonii*, and *A. haemolyticus* also could not influence mean lifespan (Fig. 2). Different from these, the lifespan curves of nematodes exposed to *Acinetobacter* strains of AC1, AC15, AC18, AC21, *A. baumannii* ATCC 19606^T^, *A. junii* NH88-14, and *A. lwoffii* DSM 2403^T^ were significantly (*P* < 0.01) different from that in control nematodes (Fig. 2). Additionally, exposure to *Acinetobacter* strains of AC1, AC15, AC18, AC21, *A. baumannii*, *A. junii*, and *A. lwoffii* significantly decreased the mean lifespan (Fig. 2). Thus, *Acinetobacter* strains of AC1, AC15, AC18, AC21, *A. baumannii*, *A. junii*, and *A. lwoffii* potentially resulted in adverse effects on lifespan of nematodes.

### Effect of exposure to different *Acinetobacter* strains isolated from the TGR region and reference strains on locomotion behavior of nematodes

**Figure 3:**
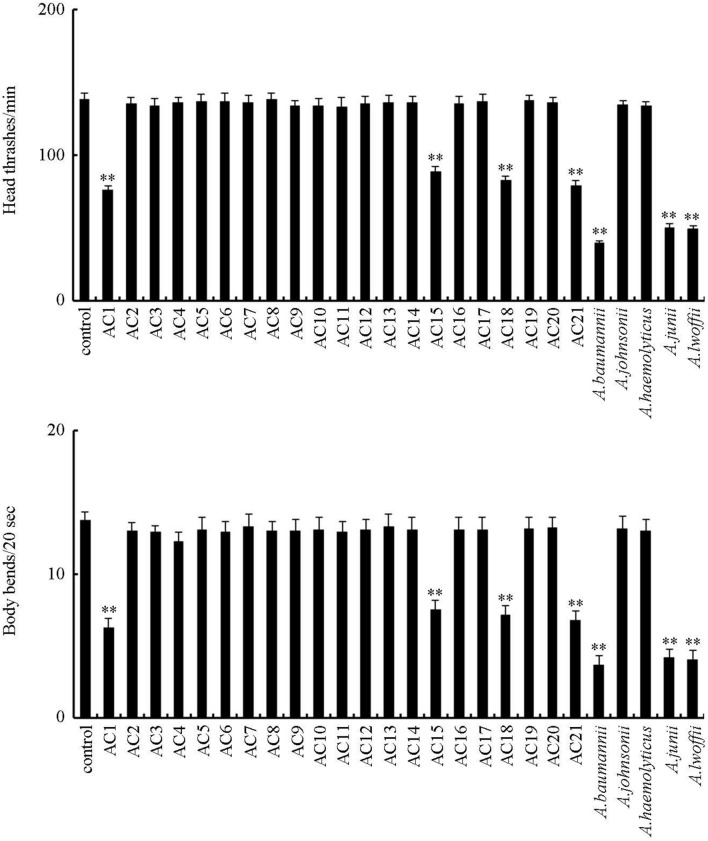
Effect of exposure to different *Acinetobacter* strains isolated from the TGR region and reference strains on locomotion behavior in wild-type nematodes. The L4-larvae nematodes were exposed to *Acinetobacter* for 24-h. Control, unexposed nematodes. Bars represent means ± SD. **P < 0.01 versus control.

Locomotion behavior is more sensitive than lifespan for assessing toxicity of environmental toxicants or stresses^25^. After exposure for 24-h, *Acinetobacter* strains of AC2, AC3, AC4, AC5, AC6, AC7, AC8, AC9, AC10, AC11, AC12, AC13, AC14, AC16, AC17, AC19, AC20, *A. johnsonii*, and *A. haemolyticus* did not obviously affect locomotion behavior (Fig. 3). In contrast, exposure to *Acinetobacter* strains of AC1, AC15, AC18, AC21, *A. baumannii*, *A. junii*, and *A. lwoffii* significantly decreased locomotion behavior (Fig. 3).

### Effect of exposure to different *Acinetobacter* strains isolated from the TGR region and reference strains in inducing activation of oxidative stress of nematodes

**Figure 4:**
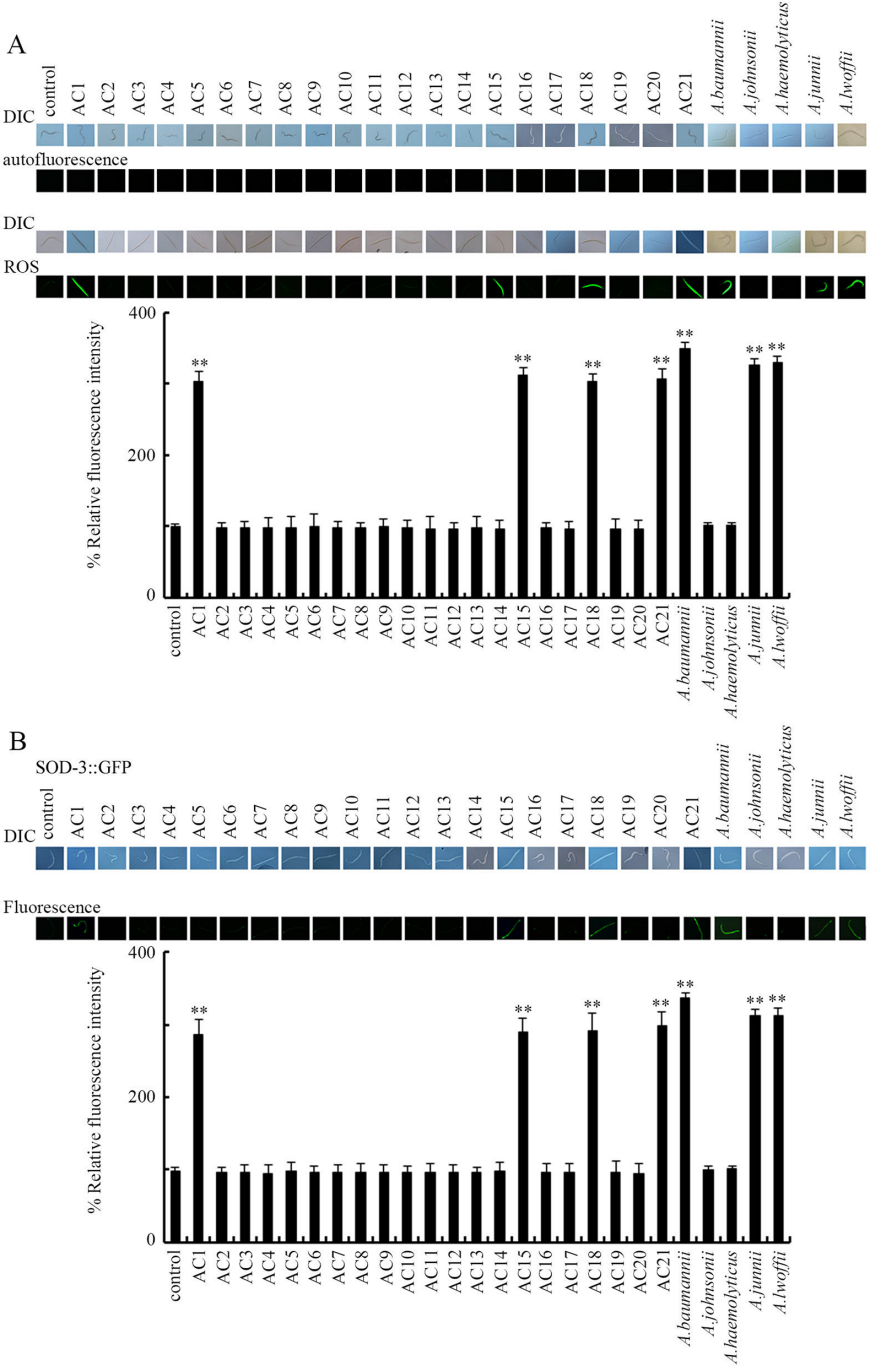
Effect of exposure to different *Acinetobacter* strains isolated from the TGR region and reference strains in inducing activation of oxidative stress in nematodes. (**A**) Effect of exposure to different *Acinetobacter* strains in inducing ROS production in wild-type nematodes. (**B**) Effect of exposure to different *Acinetobacter* strains on SOD-3::GFP expression. The L4-larvae nematodes were exposed to *Acinetobacter* for 24-h. Control, unexposed nematodes. Bars represent means ± SD. **P < 0.01 vs control.

Oxidative stress is one cellular contributor to toxicity of exposure to toxicants or stresses^25–27^. We further employed the ROS production to examine effect of *Acinetobacter* strains in inducing oxidative stress. *Acinetobacter* strains of AC1, AC15, AC18, AC21, *A. baumannii*, *A. junii*, and *A. lwoffii* for 24-h resulted in obvious induction of ROS production (Fig. 4A).

SOD-3/Mn-SOD provides a molecular basis for antioxidation defense response^25^. Moreover, we observed that exposure to *Acinetobacter* strains of AC1, AC15, AC18, AC21, *A. baumannii*, *A. junii*, and *A. lwoffii* for 24-h further led to significant increase in expression of SOD-3::GFP (Fig. 4B).

### Effect of exposure to different *Acinetobacter* strains isolated from the TGR region on expressions of antimicrobial genes in nematodes

**Figure 5:**
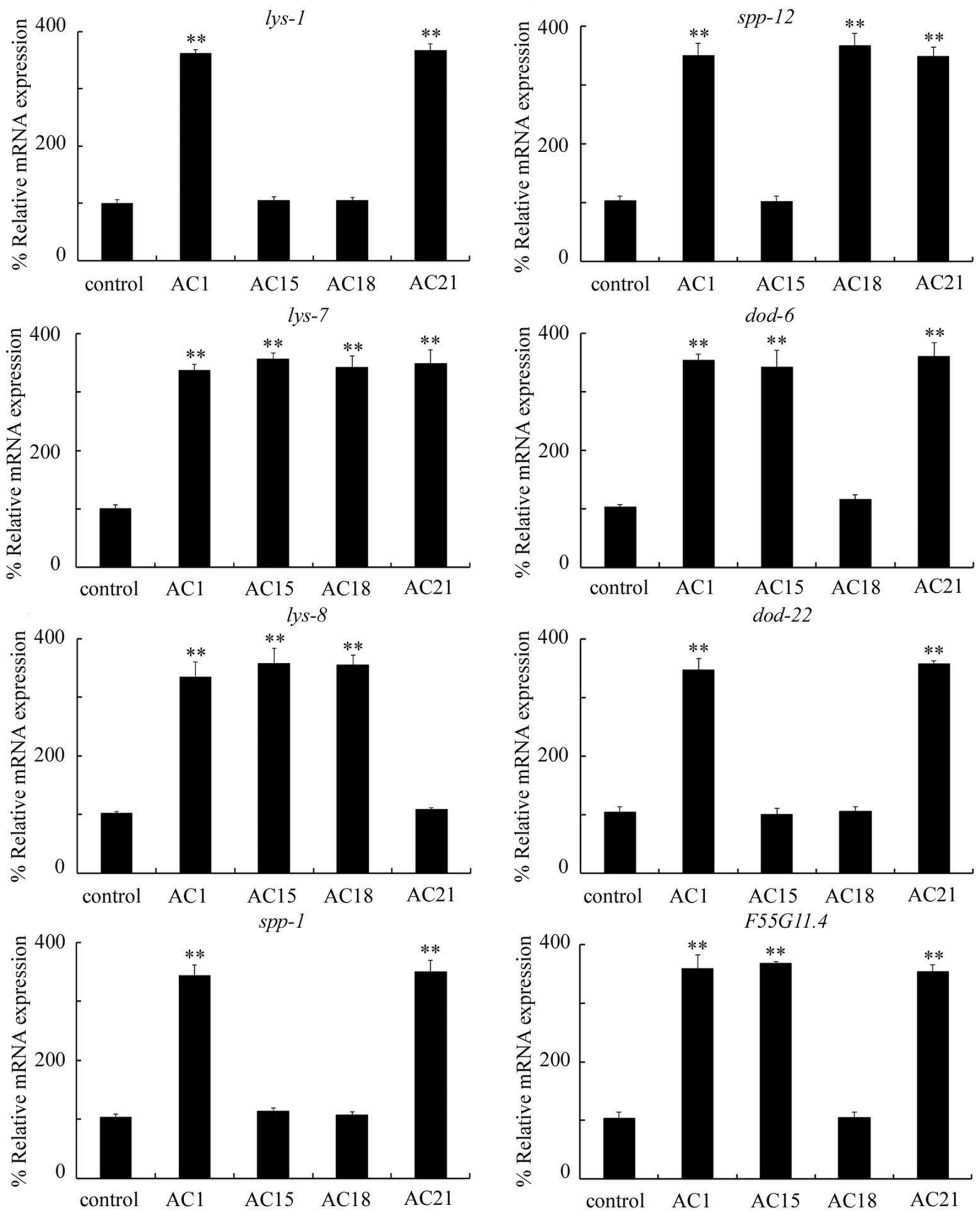
Effect of exposure to different *Acinetobacter* strains isolated from the TGR region on expressions of antimicrobial genes in wild-type nematodes. The L4-larvae nematodes were exposed to *Acinetobacter*s for 24-h. Control, unexposed nematodes. Bars represent means ± SD. **P < 0.01 versus control.

In nematodes, intestine is the important organ to activate innate immune response to pathogen infection^9^. *F55G11.4*, *dod-22*, *lys-8*, *lys-1*, *spp-12*, *lys-7*, *dod-6*, and *spp-1* are most studied intestinal anti-microbial genes^28–34^. We next selected these 8 intestinal antimicrobial genes to determine effect of different *Acinetobacter* strains isolated from the TGR region on innate immune response. The increase in these 8 intestinal antimicrobial genes function to be against pathogen infection and environmental stress^28–34^. After exposure to *Acinetobacter* strains of AC1, AC15, AC18, or AC21 for 24-h, expressions of some of these antimicrobial genes could be noticeably increased. Among these 8 antimicrobial genes, exposure to strain AC1 significantly increased the expressions of *spp-1*, *lys-8*, *lys-7*, *lys-1*, *spp-12*, *dod-6*, *dod-22*, and *F55G11.4*, exposure to strain AC15 significantly increased the expressions of *F55G11.4*, *lys-8*, *dod-6*, and *lys-7*, exposure to strain AC18 significantly increased the expressions of *lys-8*, *lys-7*, and *spp-12*, and exposure to strain AC21 significantly increased the expressions of *dod-6*, *lys-7*, *spp-12*, *lys-1*, *dod-22*, *spp-1*, and *F55G11.4* (Fig. 5). In nematodes, LYS-8, LYS-7, and LYS-1 are lysozymes, SPP-12 is a saposin-like protein, DOD-6 and DOD-22 are proteins downstream of DAF-16, SPP-1 is a caenopore, and F55G11.4 is a protein containing CUB-like domain.

### Morphological and biochemical properties of *Acinetobacter* strains of AC1, AC15, AC18, and AC21

For the *Acinetobacter* strains of AC1, AC15, AC18, and AC21, they did not show obvious difference in morphological properties of cell shape, arrangement of cell, Gram staining, and colony morphology (Table 1). The *Acinetobacter* strains of AC1, AC15, AC18, and AC21 also did not exhibit the obvious difference in biochemical properties of hydrothion, phenylalanine, gluconate, oxidase, nitrate reduction, catalase, peptone water, semi-solid agar, glucose, ornithine, raffinose, sorbitol, side calendula, and xylose (Table 1). Different from this, the *Acinetobacter* strains of AC1 and AC21 showed the negative reactions for the biochemical properties of l-arginine, l-lactic acid, d-fucose, l-histidine, l-malic acid, and d-serine (Table 1). The *Acinetobacter* strains of AC15 and AC18 exhibited the positive reactions for the biochemical properties of l-arginine, l-lactic acid, d-fucose, l-histidine, l-malic acid, and d-serine (Table 1). Additionally, the *Acinetobacter* strains of AC1 and AC21 showed the negative reactions for the biochemical properties of glucopeptone water, citrate, and gelation, whereas the *Acinetobacter* strain of AC15 exhibited the positive reactions for the biochemical properties of glucopeptone water, citrate, and gelation (Table 1).

**Table 1:** Biochemical properties of four *Acinetobacter* strains isolated from the TGR region.

Tests	AC1	AC15	AC18	AC21
Cell shape	Rod	Rod	Rod	Rod
Arrangement of cell	Pairs or single cells	Pairs or single cells	Pairs or single cells	Pairs or single cells
Gram staining	Negative	Negative	Negative	Negative
Colony morphology	Regular edges, round and smooth	Regular edges, round and smooth	Regular edges, round and smooth	Regular edges, round and smooth
Hydrothion	−	−	−	−
Phenylalanine	−	−	−	−
Gluconate	−	−	−	−
Oxidase	−	−	−	−
Nitrate reduction	−	−	−	−
Catalase	+	+	+	+
Peptone water	−	−	−	−
Glucopeptone water	−	+	−	−
Citrate	−	+	−	−
Ureophil	−	+	+	+
Semi-solid agar	−	−	−	−
Glucose	+	+	+	+
Lysine	+	+	−	−
Ornithine	−	−	−	−
Raffinose	−	−	−	−
Sorbitol	−	−	−	−
Side calendula	−	−	−	−
Xylose	−	−	−	−
Gelation	−	+	±	−
l-Arginine	−	+	+	−
l-Lactic acid	−	+	+	−
d-Fucose	−	+	+	−
l-Histidine	−	+	+	−
l-Malic acid	−	+	+	−
d-Serine	−	+	+	−

“+” stands for positive; “−” stands for negative; “±” stands for not applicable.

### Differences of main virulence genes among *Acinetobacter* strains

For understanding of differences of virulence genes from these pathogenic *Acinetobacter* strains, we checked for the presence of 14 main virulence genes (Table S4) in pathogenic strains of AC1, AC15, AC18, AC21, *A. baumannii*, *A. junii* and *A. lwoffii* and nonpathogenic strains of AC2, AC12, AC14, AC17, *A. haemolyticus*, and *A. johnsonii* by PCR. Distribution of virulence genes in tested *Acinetobacter* strains was different and pathogenic *Acinetobacter* strains generally had more virulence genes than nonpathogenic strains (Table 2). 10 or more virulence genes were detected from pathogenic strains of AC1, AC15, AC18, AC21, *A. baumannii*, *A. junii* and *A. lwoffii* (Table 2).

**Table 2:** The presence of main virulence genes in pathogenic and nonpathogenic *Acinetobacter* strains.

Strain	Pathogenicity to *C. elegans*	*ompA*	*adeF*	*bap*	*csuD*	*pgaA*	*plc1*	*lpxL*	*bfmR*	*abaI*	*emrAB*	*zigA*	*hlyD*	*omp33_36*	*barA*	Positive number
*A. baumannii*	Yes	+	+	−	+	+	+	+	+	+	+	+	+	+	+	13
*A. lwoffii*	Yes	+	−	−	+	+	+	+	−	+	−	+	+	+	+	10
*A. junii*	Yes	+	+	+	+	+	+	+	+	−	+	+	+	+	+	13
AC1	Yes	+	+	−	+	+	−	+	+	+	+	+	+	−	+	11
AC15	Yes	+	+	+	+	+	+	+	+	+	+	+	+	+	+	14
AC18	Yes	+	+	+	−	+	+	+	+	+	+	+	−	+	+	12
AC21	Yes	+	+	+	+	+	−	+	+	+	+	+	+	−	+	12
*A. haemolyticus*	No	+	+	−	−	+	−	−	+	−	+	+	+	+	+	9
*A. johnsonii*	No	−	+	−	+	−	−	+	+	+	+	+	+	+	−	9
AC2	No	−	+	−	+	+	−	+	+	+	+	+	+	+	+	11
AC12	No	+	+	+	+	+	+	+	+	+	+	+	+	+	+	14
AC14	No	+	+	−	−	−	−	−	−	−	−	+	−	−	+	4
AC17	No	+	−	−	−	−	−	−	−	−	−	+	+	−	+	4

“+” stands for positive; “−” stands for negative.

## Discussion

*Acinetobacter* has attracted significant attention because it is ubiquitous in nature and commonly found in soil, water and hospital^35^. Many *Acinetobacter* species can cause serious nosocomial infections in medicine and actively participate the nutrient cycle in the ecosystem^36^. Due to the clinical and ecological importance of *Acinetobacter*, it is proposed as a model microorganism for environmental microbiological studies, pathogenicity tests, and industrial production of chemicals^18^. Nevertheless, many research areas including biosafety, natural transformation, biodegradation, and important physiological characteristics have been limitedly investigated or neglected. We here performed a biosafety evaluation of *Acinetobacter* strains isolated from backwater area in the TGR region and 5 reference strains of *Acinetobacter* species in nematode *C. elegans.*

The high prevalence pathogens exist in the backwater area of the TGR region^7,8^. The reason to carry out the biosafety assessment of *Acinetobacter* strains is that the *Acinetobacter* has been one of dominant microorganisms in the TGR region^6^, and *Acinetobacter* isolated form the water in the TGR arises most frequently in our study. The reasons to use *C. elegans* are that it is very sensitive to various environmental exposures, and can be employed as an ideal model for the study on the pathogenesis of human pathogens, and the mechanisms in host-microbe interactions^9,13,14,16,37^. More importantly, we previously have systematically performed the biosafety evaluation of water samples from the TGR region in both flood season and quiet season^19,20^. The reasons to select 5 reference strains of *A. baumannii*, *A. lwoffii*, *A. junii*, *A. haemolyticus*, and *A. johnsonii* to expose *C. elegans* are that the genus of *Acinetobacter* comprises 68 species with validly-published names (https://apps.szu.cz/anemec/Classification.pdf, May 25, 2021) and these 5 reference speices are important clinical microorganisms^21,38^, and *A. baumannii* ATCC 19606^T^ is a model strain of pathogenic bacteria causing nosocomial infection^39^, followed by the non-*A. baumannii* species *A. haemolyticus*, *A. junii*, *A. johnsonii*, and *A. lwofii*^21^.

Our previous studies have suggested that both solid phase and liquid phase could contribute to toxicity induction of surface water sample collected from backwater areas in the TGR region^19,20^. In the liquid phase, the potential toxicants were suggested as the organic pollutants^20^. In this study, using lifespan as the toxicity assessment endpoint, we found that four (AC1, AC15, AC18, and AC21) of the isolated and examined *Acinetobacter* strains and tree reference strains of *A. baumannii*, *A. junii*, and *A. lwoffii* significantly reduced lifespan (Fig. 2). Using a more sensitive endpoint of locomotion behavior, we also observed the significant decrease in locomotion behavior after exposure to *Acinetobacter* strains of AC1, AC15, AC18, AC21, *A. baumannii*, *A. junii*, or *A. lwoffii* (Fig. 2), which further confirmed the detected toxic effect of exposure to *Acinetobacter* strains of AC1, AC15, AC18, AC21, *A. baumannii*, *A. junii*, and *A. lwoffii* on nematodes. These observations suggested that some of the *Acinetobacter* strains at the backwater area in the TGR region have the exposure risk to environmental organisms and human health. Nevertheless, not all the *Acinetobacter* strains at the backwater area in the TGR region potentially induced toxicity on environmental organisms. Our data indicated a crucial role of environmental pathogens in contributing to toxicity induction in the solid phase of water sample in backwater area in TGR region.

We further observed the significant ROS production in nematodes exposed to *Acinetobacter* strains of AC1, AC15, AC18, AC21, *A. baumannii*, *A. junii*, or *A. lwoffii* (Fig. 4A), which suggested the oxidative stress activated by exposure to these *Acinetobacter* strains. Meanwhile, we also detected the significant increase in SOD-3::GFP expression after exposure to *Acinetobacter* strains of AC1, AC15, AC18, AC21, *A. baumannii*, *A. junii*, or *A. lwoffii* (Fig. 4B), which further confirmed the oxidative stress activated by exposure to these *Acinetobacter* strains. These results suggested the close association of the toxic effects of exposure to *Acinetobacter* strains (AC1, AC15, AC18, AC21, *A. baumannii*, *A. junii*, and *A. lwoffii*) with oxidative stress activation. Nevertheless, we did not detect the decrease in SOD-3::GFP expression after exposure to the above pathogenic *Acinetobacter* strains. This may be largely due to the short exposure duration (24-h) for these pathogenic *Acinetobacter* strains. Usually, long-term exposure to toxicants at high concentrations causes decrease in SOD-3::GFP expression^9^. Exposure to nanopolystyrene (1–10 μg/L) caused increase in SOD-3::GFP expression, whereas exposure to nanopolystyrene (1000 μg/L) resulted in decrease in SOD-3::GFP expression^40^.

In nematodes, we further found that exposure to *Acinetobacter* strains of AC1, AC15, AC18, and AC21 induced increase in expressions of some antimicrobial genes (*spp-1*, *dod-22*, *lys-8*, *lys-7*, *spp-12*, *dod-6*, *lys-1*, and/or *F55G11.4*) (Fig. 5). Meanwhile, a pronounced increase in *Acinetobacter* colony-forming unit (CFU) was observed in nematodes infected with AC1, AC15, AC18, and AC21 (Fig. S1). The increase in these antimicrobial genes mediated a protective response to pathogen infection and environmental toxicants^9,28–34^. These antimicrobial genes can be expressed in the intestine (https://wormbase.org). The reason to select these intestinal antimicrobial genes is that the ROS production is mainly activated in the intestine^25^. Similarly, we also did not observe the suppression in expressions of these antimicrobial genes in nematodes exposed to *Acinetobacter* strains of AC1, AC15, AC18, or AC21, which is also largely due to the performed short exposure duration (24-h) in nematodes. Moreover, we found that exposure to *Acinetobacter* strains of AC1, AC15, AC18, and AC21 induced the different dysregulation of examined antimicrobial genes (Fig. 5). Exposure to AC1 could cause the increase in expressions of all 8 examined antimicrobial genes, and exposure to AC21 resulted in the increase in expressions of 7 examined antimicrobial genes (Fig. 5). In contrast, exposure to AC15 could cause the increase in expressions of only 4 examined antimicrobial genes, and exposure to AC18 could result in the increase in expressions of only 3 examined antimicrobial genes (Fig. 5). These results implied that *Acinetobacter* strains of AC1 and AC21 might cause the more severe toxicity at least at some aspects than *Acinetobacter* strains of AC15 and AC18.

In this study, we provide some lines of evidence to show the important value of *C. elegans* for assessing biosafety of *Acinetobacter* strains isolated from the TGR region. Nevertheless, *C. elegans* only has simple developmental structures, and dose not have some organs (such as heart, liver, lung, and kidney) observed in mammals. Therefore, the further biosafety assessment experiments in mammals for the identified four *Acinetobacter* strains are still needed.

We also examined morphological and biochemical properties of *Acinetobacter* strains of AC1, AC15, AC18, and AC21. However, we did not observe the obvious difference in morphological properties of cell shape, arrangement of cell, Gram staining, and colony morphology among the examined *Acinetobacter* strains of AC1, AC15, AC18, and AC21 (Table 1). In contrast, the observed difference in toxicity of *Acinetobacter* strains of AC1, AC15, AC18, and AC21 on nematodes might be related to the difference in some biochemical properties among the examined *Acinetobacter* strains of AC1, AC15, AC18, and AC21. For example, we observed the obvious difference in biochemical properties of l-arginine, l-lactic acid, d-fucose, l-histidine, l-malic acid, and d-serine in the *Acinetobacter* strains of AC1 and AC21 from those in the *Acinetobacter* strains of AC15 and AC18 (Table 1). To clarify if they share virulence factors that better induce to the nematode intestinal antimicrobial response, 14 main virulence genes (Table S4) of pathogenic strains of AC1, AC15, AC18, AC21, *A. baumannii*, *A. junii* and *A. lwoffii* and nonpathogenic strains of AC2, AC12, AC14, AC17, *A. haemolyticus*, and *A. johnsonii* were detected by PCR. The results showed that pathogenic *Acinetobacter* strains generally had more virulence genes than nonpathogenic strains (Table 2), and AC1 and AC21, AC15 and AC18 shared more of the same virulence genes, but nonpathogenic strains of AC2 and AC12 also had 11 and 14 virulence genes, respectively. Nevertheless, the exact underlying mechanism still needs the further careful examination.

Together, we performed a biosafety assessment of *Acinetobacter* strains isolated from backwater area in TGR region in nematodes. Among the isolated *Acinetobacter* strains, we identified four *Acinetobacter* strains with the potential to cause toxic effects on nematodes, such as the reduction in lifespan and the decrease in locomotion behavior. The observed toxic effects of *Acinetobacter* strains were associated with activation of oxidative stress. Moreover, exposure to toxic *Acinetobacter* strains caused the increase in some antimicrobial genes, suggesting the activation of innate immune response of animals against the *Acinetobacter* exposure. Considering the fact that we know little about the environmental *Acinetobacter* pathogens in the TGR region, our data provide important suggestion for exposure risk of certain *Acinetobacter* strains in the TGR region to environmental animals and human health. Our data has further implied that, after the long-term exposure, the *Acinetobacter* pathogens are potentially enriched in intestine and cause toxic effects by affecting immune response in environmental animals and human. In the future, we will further identify virulence and resistance factors and perform the sequencing for the identified four *Acinetobacter* strains isolated from the TGR region.

## Methods

### Water sampling

The water sample was collected in backwater area (N108° 23′ 25″, E30° 47′ 45″) in Wanzhou, Chongqing in the flood season^20^. The reason to select this season is that the bacterioplankton community is generally higher in this season than that in the impoundment season^6^. The detailed properties of collected surface water sample have been described previously^20^. Water sample was collected and stored as described^41^. In brief, the equal volumes (10 L) were collected from the depths of 0.5, 5, 10 m in the backwater area site. Water samples were used for the isolation of *Acinetobacter* after mixing fully in the sterile bucket, and water samples were stored at 0 °C after collection.

### *Acinetobacter* isolation, identification, and preservation

The mixed water sample was diluted serially (1, 10^−1^, and 10^−2^), inoculated into LB medium, and incubated at 37 ± 0.5 °C for 24 h. Subculture and purification of bacterial colonies were carried out by the streak plate method. For the purified bacterial isolates, genomic DNA of different bacterial isolates was extracted using bacterial genomic DNA extraction kit (TIANGEN, Beijing, China) according to the manufacturer’s instructions. The complete bacterial 16S rRNA gene was amplified with the primer set 27 F and 1492R. PCR products were visualized using 1% agarose gels stained with ethidium bromide. Positive amplicons were quantified using a PicoGreen dsDNA Assay kit (Invitrogen, CA, USA). Purified products were sequenced and analyzed by Magigen (Guangzhou Magigen Biotechnology Co., Ltd., China). Phylogenetic tree was constructed using the Mega 5.0 program using the neighbor-joining (N-J) method with a 1000-bootstrap.

All identified *Acinetobacter* strains were preserved by freeze drying^42^. The exponential phase cells of *Acinetobacter* strains grown in LB medium for 18 h were suspended in aseptic no-fat skimmed milk with an initial cell concentration of 10^8^–10^9^ CFU/mL. The bacterial mixture within ampoules vials was frozen at − 20 °C for 2 h, followed by − 80 °C for 12 h. After that, they were loaded onto the freeze dryer. Both primary drying and secondary drying for 25 h after the freezing were performed. The freeze-dried products were packaged in blister packs and stored in the refrigerator at − 80 °C.

When needed, freeze-dried powders were diluted with sterilized water, and then the suspensions were streak-inoculated onto a LB medium using an inoculation loop. A single colony was inoculated into sterilized LB broth and the bacteria grew to the log phase in a constant temperature oscillator at 37 °C for the use^43,44^.

### Reference strains of *Acinetobacter* species

*A. baumannii* (ATCC 19606^T^), *A. lwoffii* (DSM 2403^T^), *A. junii* (NH88-14), *A. haemolyticus* (TTH0-4), and *A. johnsonii* (H10) from China General Microbiological Culture Collection Center (CGMCC) were used to expose the *Caenorhabditis elegans* in this study. The information of these reference strains is listed in the Table S2.

### Analysis of *Acinetobacter* properties

Different *Acinetobacter* strains inoculated on broth agar medium were incubated for 24 h at 37 °C^45^. Primary identification and characterization of different *Acinetobacter* strains were performed to determine cell shape, arrangement of cell, gram staining, and colony morphology using UVsolo 2 touch (Analytik Jena AG, Germany)^46^. After the growth at 37 °C for 24 h, the biochemical properties of different *Acinetobacter* strains were further determined using standard Enterobacteriaceae biochemical identification tube (HANGWEI, Hangzhou Microbiology Reagent Co., Ltd)^47^.

### Maintenance of *C. elegans*

CF1553/*muIs84*[SOD-3:GFP] and wild-type N2 were used. Normal nematode growth media (NGM) plates were used to maintain nematodes^48^. To prepare synchronized L4-larvae, gravid worms were first treated with bleaching solution (0.45 M NaOH and 2% HOCl)^49^. The released eggs were let to further develop into the L4-larvae population.

### *Acinetobacter* pathogenesis assay

The L4-larvae population was exposed to different *Acinetobacter* strains. Different *Acinetobacter* strains were seeded on modified NGM containing 0.35% peptone. Exposure to different *Acinetobacter* strains was started by transferring nematodes onto each assay plate by adding 60 animals to each assay NGM plate. Full-lawn assay plate was used for *Acinetobacter* pathogenesis assay as described^50^. That is, the surface of assay NGM plate was all seeded *Acinetobacter* strains. The aim of using full lawn assay was to exclude the possibility of effect from the avoidance behavior of nematodes to *Acinetobacter* strains.

### *Acinetobacter* CFU analysis

The method was performed basically as described^51^. After the infection, the treated nematodes were transferred to M9 solution containing 25 mM levamisole to paralyze the nematodes. Nematodes were first transferred to a NGM plate containing ampicillin (1 mg/mL) and gentamicin (1 mg/mL) for 15 min to eliminate *Acinetobacter* on the body surface. Nematodes were further transferred to a new NGM plate containing ampicillin (1 mg/mL) and gentamicin (1 mg/mL) for 30 min to remove any residual *Acinetobacter*. After that, the nematodes were lysed with a motorized pestle. The lysates were serially diluted in M9 solution and plated on Luria–Bertani plates. After overnight incubation at 37 °C, the colonies were counted to determine the CFU per nematode. The experiments are repeated for three times.

### Lifespan assay

After exposure of L4-larvae nematodes to different *Acinetobacter* strains for 24-h, the survival of worms was counted every day at 20 °C^52^. If no response was observed after prodding using platinum wire, the worms were considered as dead. The animals were transferred daily during the first 7-day. For the lifespan assay, 60 animals were examined for each treatment. Three replicates were carried out. We used log-rank test to analyze the lifespan curve data. Survival curves were considered to have significant difference if *P *values were ≤ 0.01.

### Locomotion behavior

Locomotion behavior reflects the functional state of motor neurons^53^. Body bend and head thrash were selected as the endpoints^54^. After exposure, the worms were first washed with M9 buffer. After that, assuming that animals traveled along x axis, a body bend is defined as a change of bending direction at the mid-body. A head thrash is defined as a change of posterior bulb direction along y axis. For each treatment, 40 animals were analyzed.

### Activation of oxidative stress

Production of reactive oxygen species (ROS) reflects the activation of oxidative stress^55^. The method was performed as described^56^. After the exposure to different *Acinetobacter* strains, the animals were labeled for 3 h using CM-H_2_DCFDA (1 µM). After that, the animals were observed at 488 nm (excitation wavelength)/510 nm (emission filter) under a laser scanning confocal microscope. Using Image J software, we semi-quantified intestinal fluorescence intensity in comparison to intestinal autofluorescence. For each treatment, 50 animals were examined.

In nematodes, *sod-3* encodes mitochondrial Mn-SOD^9^. Using Image J software, fluorescence intensity of SOD-3::GFP signals in the intestine was semi-quantified. For each treatment, 50 animals were examined.

### Quantitative real-time polymerase chain reaction (qRT-PCR)

The total RNAs of control and exposed nematodes were extracted using Trizol. Using a spectrophotometer, concentration and purity of the obtained RNAs were determined. We performed the reverse transcriptase reaction with Mastercycler gradient PCR system for cDNA synthesis. With the aid of SYBR Green qRT-PCR master mix, transcriptional expression of *spp-1*, *lys-8*, *lys-7*, *lys-1*, *spp-12*, *dod-6*, *dod-22*, and *F55G11.4* were determined in real-time PCR system. The reference gene was *tba-1*. Three biological replicates were carried out. Primer information is provided in Table S3.

### Polymerase chain reaction (PCR) of virulence genes

Primers were designed according to the published sequences of virulence genes of *Acinetobacter* on GenBank. Primer information was provided in Table S4. All experimental strains were inoculated onto beef extract agar and cultured at 37 ℃ for 24 h. A single colony was selected and inoculated in beef extract broth at 37 ℃ for 12 h, and the bacterial culture was used as the template. PCR was undertaken in a final volume of 25 μL using the PCR kit (Sangong Bioengineering (Shanghai) Co., Ltd.) with 1μL of each primer and 1 μL of the template. The thermal cycling parameters were 30 s at 98 °C for, followed 35 cycles of 5 s at 98 °C, 5 s at 59 °C and 60 s at 72 °C. 5μL PCR products were analyzed on agarose 1.4% (w/v) gels.

### Statistical analysis

Statistical analysis was carried out using SPSS Statistics 19.0 Software (SPSS Inc., USA.). Probability level of 0.01 was considered statistically significant. Using one-way analysis of variance (ANOVA), the differences between groups were tested.

## Supplementary Information

Supplementary Information.
