# Supplementary material for: Biosafety assessment of Acinetobacter strains isolated from the Three Gorges Reservoir region in nematode Caenorhabditis elegans
Source: Sci Rep. 2021 Oct 5;11:19721. doi: 10.1038/s41598-021-99274-0 (PMC8492797; doi:10.1038/s41598-021-99274-0)
Supplement: Supplementary file 1 — Supplementary Information. [file 41598_2021_99274_MOESM1_ESM.doc]

**Biosafety assessment of *Acinetobacter* strains isolated from the Three Gorges Reservoir region in nematode** ***Caenorhabditis elegans***

Yunjia Deng^1, 2^, Huihui Du^1, 3^, Mingfeng Tang^1, 2^, Qilong Wang^1, 3^, Qian Huang^1^, Ying He^1, 2^, Fei Cheng^1, 2^, Feng Zhao^1, 2^, Dayong Wang^1^ & Guosheng Xiao^1, 2, 3, *^

^1^College of Biology and Food Engineering, Chongqing Three Gorges University, Wanzhou, Chongqing, China

^2^Key Laboratory of Water Environment Evolution and Pollution Control in Three Gorges Reservoir, Chongqing Three Gorges University, Wanzhou, Chongqing, China

^3^Engineering Technology Research Center of Characteristic Biological Resources in Northeast Chongqing, Chongqing Three Gorges University, Wanzhou, Chongqing, China

*Corresponding author.

E-mail address: [xgs03@sanxiau.edu.cn](mailto:xgs03@sanxiau.edu.cn) (G. Xiao)

**Supporting Information:**


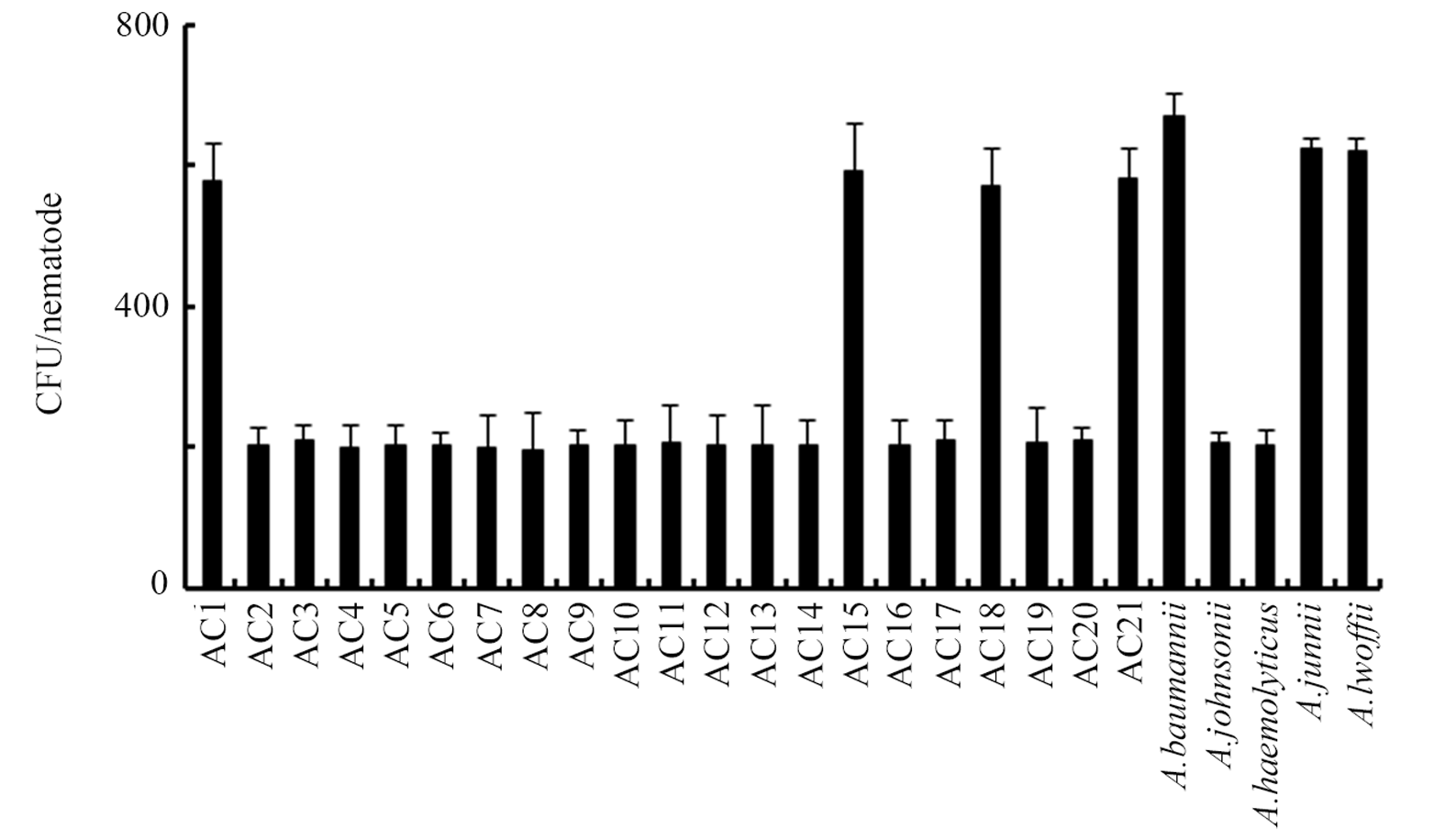


**Figure S1.** Comparison of *Acinetobacter* colony-forming unit (CFU) for different *Acinetobacter* strains isolated from the TGR region and reference strains in wild-type nematodes. The L4-larvae nematodes were exposed to *Acinetobacter* for 24-h.

**Table S1.** The used 21 *Acinetobacter* strains isolated from the TGR region

| No. | Isolates | Accession no. | 16s rRNA sequence length (bp) | Homologous strains | Accession no. | 16s rRNA sequence length (bp) | Query cover | Identity |
| --- | --- | --- | --- | --- | --- | --- | --- | --- |
| 1 | *Acinetobacter sp.* AC1 | MW386206 | 1398 | *Acinetobacter sp.* FZKA6 | KU193772 | 1403 | 100% | 99.86% |
| 2 | *Acinetobacter sp.* AC2 | MW386207 | 1400 | *Acinetobacter sp.*TS39 | EU073105 | 1413 | 100% | 99.93% |
| 3 | *Acinetobacter sp.* AC3 | MW386208 | 1398 | *Acinetobacter uncultured Acinetobacter sp.* | HE575551 | 1501 | 99% | 96.57% |
| 4 | *Acinetobacter sp.* AC4 | MW386209 | 1397 | *Acinetobacter sp.* TS39 | EU073105 | 1413 | 99% | 99.36% |
| 5 | *Acinetobacter sp.* AC5 | MW386210 | 1397 | *Acinetobacter uncultured Acinetobacter sp.* | HE575551 | 1501 | 100% | 96.57% |
| 6 | *Acinetobacter sp.* AC6 | MW386211 | 1398 | *Acinetobacter uncultured Acinetobacter sp.* | HE575551 | 1501 | 100% | 96.57% |
| 7 | *Acinetobacter sp.* AC7 | MW386212 | 1397 | *Acinetobacter sp.*TS39 | EU073105 | 1413 | 100% | 99.86% |
| 8 | *Acinetobacter* *sp.* AC8 | MW386213 | 1400 | *Acinetobacter* *uncultured Acinetobacter sp.* | HE575551 | 1501 | 100% | 96.58% |
| 9 | *Acinetobacter sp.* AC9 | MW386214 | 1397 | *Acinetobacter sp.*TS39 | EU073105 | 1413 | 100% | 99.64% |
| 10 | *Acinetobacter sp.* AC10 | MW386215 | 1397 | *Acinetobacter uncultured Acinetobacter sp.* | HE575551 | 1501 | 99% | 96.56% |
| 11 | *Acinetobacter sp.* AC11 | MW386216 | 1399 | *Acinetobacter uncultured Acinetobacter sp.* | HE575551 | 1501 | 100% | 96.43% |
| 12 | *Acinetobacter sp.* AC12 | MW386217 | 1398 | *Acinetobacter uncultured Acinetobacter sp.* | HE575551 | 1501 | 100% | 96.36% |
| 13 | *Acinetobacter sp.* AC13 | MW386218 | 1399 | *Acinetobacter sp.* B113 | EU883929 | 1408 | 99% | 99.93% |
| 14 | *Acinetobacter sp.* AC14 | MW386219 | 1402 | *Acinetobacter uncultured Acinetobacter sp.* | HE575551 | 1501 | 99% | 95.38% |
| 15 | *Acinetobacter johnsonii* AC15 | MW386220 | 1399 | *Acinetobacter johnsonii ATCC 17909* | HE651920 | 1530 | 99% | 99.57% |
| 16 | *Acinetobacter haemolyticus* AC16 | MW386221 | 1400 | *Acinetobacter haemolyticus ATCC 17906* | HE651915 | 1530 | 100% | 98.22% |
| 17 | *Acinetobacter sp.* AC17 | MW386222 | 1398 | *Acinetobacter uncultured Acinetobacter sp.* | HE575551 | 1501 | 100% | 96.57% |
| 18 | *Acinetobacter sp.* AC18 | MW386223 | 1399 | *Acinetobacter sp.*TS39 | EU073105 | 1413 | 100% | 99.86% |
| 19 | *Acinetobacter sp.* AC19 | MW386224 | 1395 | *Acinetobacter uncultured Acinetobacter sp.* | HE575551 | 1501 | 99% | 96.21% |
| 20 | *Acinetobacter sp.* AC20 | MW386225 | 1398 | *Acinetobacter uncultured Acinetobacter sp.* | HE575551 | 1501 | 99% | 96.21% |
| 21 | *Acinetobacter sp.* AC21 | MW386226 | 1399 | *Acinetobacter sp.* P2-2 | EU276092 | 1502 | 99% | 98.00% |

***Table S2.*** *Five reference strains of Acinetobacter species* *from China General Microbiological Culture Collection Center (CGMCC)*

|  | Reference strain | CGMCC number | Strain number | Strain source | Isolation source | Model strain | Accession no. |
| --- | --- | --- | --- | --- | --- | --- | --- |
| 1 | *Acinetobacter baumannii* | 1.6769 | ATCC 19606 | Japan collection of microorganisms (JCM) | Urine | Yes | HE651907 |
| 2 | *Acinetobacter lwoffii* | 1.2005 | DSM 2403 | Polish Collection of Microorganisms | Human skin | Yes | X81665 |
| 3 | *Acinetobacter junii* | 1.8764 | NH88-14 | Ningbo University, China | Pond water for *Portunus trituberculatus* | No | FJ447529 |
| 4 | *Acinetobacter haemolyticus* | 1.12996 | TTH0-4 | Cold and Arid Regions Environmental and Engineering Research Institute, Chinese Academy of Sciences | Soil | No | KF704077 |
| 5 | *Acinetobacter johnsonii* | 1.8823 | H10 | Chengdu Institute of Biology, Chinese Academy of Sciences | Well water | No | FJ009371 |

**Table S3.** Primer information for qRT-PCR

| Genes | Forward primer (5’-3’) | Reverse primer (5’-3’) |
| --- | --- | --- |
| *lys-1* | TTCGGATCTTTCAAGAAGGC | TGGGATTCCAACAACGTAAA |
| *spp-12* | CTAATGGTTGTGCCCGCAAT | AGCCAGACATCGAGAGCAAC |
| *lys-7* | CTGCCATTCGGCATCAGTCA | GCACAATAACCCGCTTGTTT |
| *dod-6* | CGTCGCCCTCTACTTCCT | ACGGCACAGTGACCATCT |
| *lys-8* | TCAGTCTCCGTCAAGGTC | GAAGCTGGCTCAATGAAA |
| *dod-22* | CCAGGATACAGAATACGT | CCAGAGATGACTTCAGTT |
| *spp-1* | GCATCACGGTGTTTTCTGTG | GCAACAGCATAGTCCAGCAA |
| *F55G11.4* | GGATCCGTGTATTTGGCT | GTGAAGACATATGTGCTC |
| *tba-1* | TCAACACTGCCATCGCCGCC | TCCAAGCGAGACCAGGCTTCAG |

**Table S4.** Information of virulence genes of *Acinetobacter* and primers for PCR

| Virulence genes | Functions | Primer sequences | Accession no. | Product length（pb） | Tm (℃) |
| --- | --- | --- | --- | --- | --- |
| *ompA* (outer membrane protein A) | Antibiotic- and serum-resistance, biofilm formation, | F: TTTCCAAGACAGCCAACACAACAATG | KJ363323.1 | 371 | 59 |
|  |  | R: AAGCACCAACACCAGCGTTACC |  |  |  |
| *omp33-36* (porin Omp33-36) | Induction of apoptosis and modulation of autophagy | F: CATACGTTCCAACTCCTTACCTTCCTG | NZ_CP043953.1 | 228 | 59 |
|  |  | R: CAGCAGAGTAGATACCGTTACCGATG |  |  |  |
| *csuD* (Csu fimbrial usher) | Biofilm formation | F: CTTCAACCGCTCTGTTCCGTCTG | AP022836.1 | 349 | 59 |
|  |  | R: CGAATAGTAAGGCGTCACCGATGG |  |  |  |
| *plc1* (phospholipase C1) | Lipolytic activity for iron acquisition | F: GATTCGTGTCGCTCAAGAACTATTGC | NC_010611.1 | 255 | 59 |
|  |  | R: TGTTTGCCCGCCTCAACCATATAG |  |  |  |
| *lpxL* (LpxL/LpxP family Kdo(2)-lipid IV(A) lauroyl/palmitoleoyl acyltransferase ) | Immune evasion | F: TGCGTGGTTTGATTCGTCATCTTAAAG | NC_010611.1 | 273 | 59 |
|  |  | R: GCGTTGCATCATCGACTTCATCTTC |  |  |  |
| *emrAB* (multidrug efflux transporter EmrAB transcriptional repressor) | Osmotic stress resistance | F: ATCGCACTTGAGTCTTCTGGTTTCC | CP015121.1 | 306 | 59 |
|  |  | R: ACCTAAAGTATGACCGCCAGCAATC |  |  |  |
| *abaI* (acyl-homoserine-lactone synthase) | Virulence, motility, conjugation | F: GGGAGTTGAACTGTCCAAACAATGAAG | NC_010611.1 | 254 | 59 |
|  |  | R: CTGACTGCTAGAGGAAGGTGGATTTG |  |  |  |
| *zigA* (zinc metallochaperone GTPase) | Zinc metallo-chaperone | F: TGGGCGAGCAAGATGAAAGAACTG | NZ_CP015121.1 | 394 | 59 |
|  |  | R: AAGCCTTTGGAACGCACGACTC |  |  |  |
| *barA* (acinetobactin export ABC transporter permease/ATP-binding subunit) | Iron intake | F: GCCATCGTGACACCTTCGCTAC | NC_010611.1 | 257 | 59 |
|  |  | R: CCAACTCCACTCGGTCCTGTAATTG |  |  |  |
| *bfmR* (response regulator transcription factor) | QS-regulated two-component system involved in biofilm formation | F: TCGCCCACACTATCATCAACCAATC | NC_010611.1 | 264 | 59 |
|  |  | R: GTAAAGTCAACAAGCTCACCGTTCAAC |  |  |  |
| *adeF* (multidrug efflux RND transporter periplasmic adaptor subunit) | Biofilm formation | F: CCCAGCGATTCTGATTAGTCCAACC | KR297239.1 | 226 | 59 |
|  |  | R: CGGAACGAGATGCGGTGTAACAG |  |  |  |
| *bap* (biofilm-associated protein) | Biofilm formation | F: GGTACTCCAGCAACGGTTGTAGG | KT900138.1 | 235 | 59 |
|  |  | R: GTGTAGCATCTGCCGAAGGATCTG |  |  |  |
| *pgaA* (poly-beta-1,6 N-acetyl-D-glucosamine exporter porin) | Biofilm formation | F: GAATGGCAGGCGAACCGTAAGG | FJ866497.1 | 370 | 59 |
|  |  | R:CTTGGCTGTTAGTACCGTAGAAACCTC |  |  |  |
| *hlyD* (secretion protein) | Secretion of proteins involved in biofilm formation | F: GCTCACAGTTACTCTACCTCATTCCAC | NC_016603.1 | 290 | 59 |
|  |  | R: GCCAGGGCGTAAGTGTTCTATTCC |  |  |  |
